# Stoichiometric evolutions of PH_3_ under high pressure: implication for high-*T*_c_ superconducting hydrides

**DOI:** 10.1093/nsr/nwz010

**Published:** 2019-01-22

**Authors:** Ye Yuan, Yinwei Li, Guoyong Fang, Guangtao Liu, Cuiying Pei, Xin Li, Haiyan Zheng, Ke Yang, Lin Wang

**Affiliations:** 1 Center for High Pressure Science and Technology Advanced Research, Shanghai 201203, China; 2 School of Physics and Electronic Engineering, Jiangsu Normal University, Xuzhou 221116, China; 3 Key Laboratory of Carbon Materials of Zhejiang Province, College of Chemistry and Materials Engineering, Wenzhou University, Wenzhou 325035, China; 4 Department of Physics, Fudan University, Shanghai 200433, China; 5 Shanghai Institute of Applied Physics, Chinese Academy of Sciences, Shanghai 201203, China

**Keywords:** high pressure, hydrides, superconductivity, stoichiometric evolution

## Abstract

The superconductivity of hydrides under high pressure has attracted a great deal of attention since the recent observation of the superconducting transition at 203 K in strongly compressed H_2_S. It has been realized that the stoichiometry of hydrides might change under high pressure, which is crucial in understanding the superconducting mechanism. In this study, PH_3_ was studied to understand its superconducting transition and stoichiometry under high pressure using Raman, IR and X-ray diffraction measurements, as well as theoretical calculations. PH_3_ is stable below 11.7 GPa and then it starts to dehydrogenate through two dimerization processes at room temperature and pressures up to 25 GPa. Two resulting phosphorus hydrides, P_2_H_4_ and P_4_H_6_, were verified experimentally and can be recovered to ambient pressure. Under further compression above 35 GPa, the P_4_H_6_ directly decomposed into elemental phosphorus. Low temperature can greatly hinder polymerization/decomposition under high pressure and retains P_4_H_6_ up to at least 205 GPa. The superconductivity transition temperature of P_4_H_6_ is predicted to be 67 K at 200 GPa, which agrees with the reported result, suggesting that it might be responsible for superconductivity at higher pressures. Our results clearly show that P_2_H_4_ and P_4_H_6_ are the only stable P–H compounds between PH_3_ and elemental phosphorus, which is helpful for shedding light on the superconducting mechanism.

## INTRODUCTION

Since superconducting mercury was first reported [1,2], scientists have continued to search for new high critical temperature (*T*_c_) materials. In 2004, Ashcroft studied hydrogen-dominant hydrides [3], in which condensed H_2_ may contribute to a high *T*_c_. Motivated by this work, extensive theoretical investigations on this system have been reported, such as SiH_4_ [4], GeH_4_ [5], GaH_3_ [6], SiH_4_(H_2_)_2_ [7], CaH_6_ [8] and YH_6_ [9], etc. A few remarkable high-*T*_c_ materials have also been observed in subsequent experimental studies. Recently, Drozdov *et al.* reported the superconductive transition of H_2_S at 203 K and 155 GPa [10], which broke the highest *T*_c_ record [11]. Many theoretical [12,13] and experimental [14] studies have explored its stoichiometry and structure, which play an important role in understanding the underlying mechanism of superconductivity.

Very recently, PH_3_, a typical hydrogen-rich hydride, has attracted a great deal of research interest because of its superconducting transition discovered by Drozdov and his co-workers [15–20]. Their experimental work revealed that PH_3_ might be a high-temperature superconducting candidate. From the resistance measurements, a superconducting transition signature was observed at *T*_c_ of 30 K. This increased to 103 K with pressures up to 207 GPa. However, structural information was not provided, and the origin of the superconducting transition remains puzzling. Subsequent theoretical studies [16–19] showed that the P–H compound should also be a complex system, and all the predicted structures were metastable with respect to the elemental phase.

Flores-Livas *et al.* [16] studied the phase diagram of phosphorus hydrides with different stoichiometries and found that they tended to decompose into phosphorus and hydrogen at high pressure. Liu *et al.* [17] predicted a PH_3_ phase with a monoclinic structure (C2/m) and a *T*_c_ of 83 K at 200 GPa, which is closer to the observed superconducting transition temperature. Shamp *et al.* [18] predicted that PH_3_ is thermodynamically unstable during decomposition into the elemental phases, as well as PH_2_ and H_2_. Two PH_2_ phases with C2/m and I4/mmm symmetry were computed as metastable at 200 GPa. The corresponding superconducting critical temperatures were 76 and 70 K, respectively. Bi *et al.* [19] found that a dynamically stable PH_2_ phase was the best according to the observed superconducting transition at 80 GPa. The PH_3_ phase to PH_2_ phase reaction was exothermic at that pressure, which proves the spontaneity of the reaction.

Until now, the PH_3_ phase under compression has remained unknown and no relevant experimental studies have been reported. The high-pressure stoichiometry and structural behavior of PH_3_ are critical to understanding the superconducting transition in the P–H system, which needs to be experimentally determined. For this purpose, we studied the structural behavior of PH_3_ under high pressure. We identified the pressure-induced step-by-step polymerization of PH_3_ and a route to elemental phosphorus that unveiled the unknown transition process and provides experimental evidence for understanding the underlying mechanism of the superconductivity of P–H compounds.

## RESULTS AND DISCUSSION

### Stoichiometric evolutions of PH_3_ at room temperature

After the PH_3_ gas was loaded into the sample chamber of the diamond anvil cell (DAC) and returned to room temperature, a colorless and transparent sample (Supplementary Fig. 1, available as Supplementary Data at *NSR* online) was observed. The characteristic Raman peaks (Supplementary Fig. 1, available as Supplementary Data at *NSR* online) at 978 (υ_2_, symmetric bending mode), 1104 (υ_4_, asymmetric bending mode), 2317 (υ_1_, stretching mode) and 2331 (υ_3_, stretching mode; shoulder) cm^−1^ agreed well with previous reports [21], indicating the existence of PH_3_ in the chamber.

**Figure 1.**
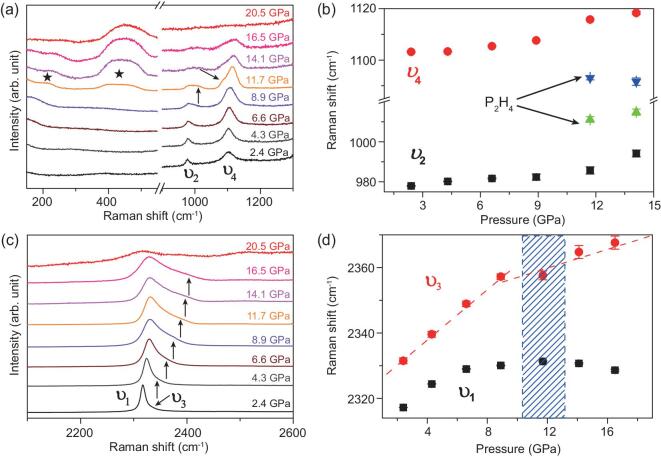
(a, c) Raman spectra of PH_3_ at various pressures at room temperature. The peak positions of υ_2_, υ_4_ (b) and υ_1_, υ_3_ (d) as functions of pressure.

The X-rays can damage the sample (Supplementary Fig. 2, available as Supplementary Data at *NSR* online), so Raman and infrared absorption spectroscopy (IR) were mainly used for our *in situ* studies of PH_3_ at high pressure. Figure 1a and c shows the Raman spectra of the sample during compression. Under high pressure, these characteristic modes blue shifted and broadened (Fig. 1b and d) and eventually vanished at 20.5 GPa. Several new peaks (marked by black asterisks and arrows inFig. 1a) were observed at around 11.7 GPa, which suggested a phase transition. For the P–H stretching modes, we also noticed a dramatic expansion of the characteristic bonds. Figure 1d shows the peak positions of the υ_1_ and υ_3_ modes as a function of pressure. The peak shift of υ_1_ dramatically decreased and started to red shift at 11.7 GPa. We attributed these changes to a transition in the sample near 11.7 GPa.

These new peaks in the Raman spectra (Fig. 1a) were consistent with previous studies about P_2_H_4_ at ambient pressure. The two new peaks at low frequencies correspond to the PH_2_ rocking mode and P–P stretching mode in the P_2_H_4_ molecule, which were observed at around 217 and 436 cm^−1^, respectively [22,23]. The emergency P–P bond at 11.7 GPa proved the dimerization of PH_3_ molecules. The other new peaks at 1007 and 1093 cm^−1^ were from the PH_2_ scissoring modes in the P_2_H_4_ molecule, which also agrees with previous reports. These factors suggest that the pressure-induced transition is due to the dimerization of PH_3_ at high pressure.

**Figure 2.**
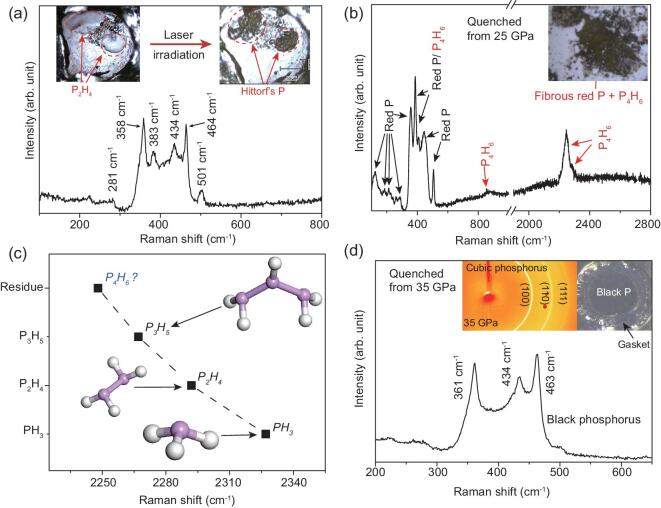
(a) The Raman spectrum of the Hittorf's phosphorus transformed from the liquid sample after laser irradiation. The inset images show the photo-induced transition of the liquid residue before and after laser irradiation. (b) The Raman spectrum of the sample decompressed from 25 GPa. The inset picture shows the optical micrograph of the decompressed sample. (c) The frequency trend of the P–H stretching in P_n_H_n+2_ (*n* = 1, 2, 3 and 4). (d) The Raman spectrum of the sample quenched from 35 GPa. The inset shows the XRD pattern of the sample at 35 GPa and the optical micrograph of the decompressed sample.

To verify the dimerization, we also studied the decompressed sample. A liquid sample was obtained after quenching to ambient conditions, as shown in the microphotograph of the decompressed sample (inset optical images in Fig. 2a). It is well known that P_2_H_4_ is a liquid at ambient pressure [22,24], which confirms that pressure drives the dimerization of PH_3_ to form P_2_H_4_ via this reaction:

(1) \begin{equation*} {\rm{2PH}_3} \to {{\rm{P}}_{\rm{2}}}{{\rm{H}}_{\rm{4}}}{\rm{ + }}{{\rm{H}}_{\rm{2}}.} \end{equation*}

We further employed Raman to measure the recovered liquid sample. However, after laser irradiation, the liquid sample decomposed and generated Hittorf's phosphorus [25,26] (Fig. 2a) according to the photodecomposition properties of P_2_H_4_ [24]. This offers more evidence of our findings.

We also employed IR to trace the *in situ* information of the new product at high pressure. Supplementary Fig. 3a, available as Supplementary Data at *NSR* online, shows the IR peak near 1095 cm^−1^ broadened and shifted slightly to a lower frequency with increasing pressure, but an obvious new shoulder was observed at around 1058 cm^−1^ after decompressing the sample to 11.8 GPa (Supplementary Fig. 3c and d, available as Supplementary Data at *NSR* online). This new shoulder matched the P_2_H_4_ scissors mode well, which was observed at around 1052 cm^−1^ in a solid state at ambient pressure [27]. This characteristic mode confirms the existence of P_2_H_4_. In addition to the P–H stretching modes in the IR spectra (Supplementary Fig. 3b, available as Supplementary Data at *NSR* online), a new shoulder at around 2329 cm^−1^ was observed, and it became stronger and stronger with increasing pressure. After it had quenched to 11.8 GPa, the new shoulder peak was more obvious compared to the IR spectrum measured at 12 GPa during compression. This proves dimerization.

As the pressure increased, the P_2_H_4_ showed piezochromism. It became yellow, then red and darkened, and eventually became opaque at pressures higher than 25 GPa, consistently with the observations of Drozdov *et al.* at low temperatures (180 K). As the sample became totally opaque, the vibrational signal vanished and hindered the *in situ* high-pressure vibrational spectra measurement. Therefore, we had to quench the sample to ambient conditions from different pressures (25 and 35 GPa) and employed Raman spectroscopy to investigate the different quenched residues. Interestingly, once the sample became completely opaque above 25 GPa, it maintained its opaque solid state even when decompressed to room pressure. This irreversible process suggests that a new transition occurred at higher pressures.

Figure 2b shows that the Raman spectrum of the residue quenched from 25 GPa after the opaque transition. A weak peak near 873 cm^−1^ belonging to PH_2_ twisting and a strong peak at 2248.5 cm^−1^ belonging to P–H stretching exist in the spectrum. This new P–H stretching peak is located at a much lower wave number than in PH_3_, P_2_H_4_ (∼2292 cm^−1^) and P_3_H_5_ (∼2267 cm^−1^) [24], suggesting that the residue contained a new kind of phosphorus hydride. Figure 2c shows the P–H stretching mode of P*_n_*H*_n_*_+2_ shifts to lower frequency as *n* becomes larger. Following this trend, we deduced that the new phosphorus hydride was P_4_H_6_, which suggests that the P_2_H_4_ molecules continued to dimerize and form P_4_H_6_ at higher pressure.

To confirm the second dimerization, we calculated the Raman modes of P_4_H_6_ using the Gaussian 09 program at the B3LYP/6–311(d, p) level [28]. Supplementary Table 1, available as Supplementary Data at *NSR* online, shows the calculated Raman modes of two typical P_4_H_6_ conformers, in which the four phosphorus atoms are linear and U-type (Supplementary Fig. 4a and b, available as Supplementary Data at *NSR* online). The calculated Raman spectra show that they both have four characteristic bands corresponding to the stretching vibration (350–450 cm^−1^) of the P–P bond, twisting vibration (700-900 cm^−1^) of the PH_2_ group, scissoring vibration (∼1070 cm^−1^) of the PH_2_ group and stretching vibration of the P–H bond, respectively. Moreover, the P–H stretching mode can further shift to a lower frequency (2278 cm^−1^). From Supplementary Table 1, available as Supplementary Data at *NSR* online, we can see that the P–P stretching bonds and the twisting vibration of the PH_2_ group from linear P_4_H_6_ are closer to our observed peak, suggesting that the linear type P_4_H_6_ is the more possible conformer in the residue.

Besides the peaks from P_4_H_6_, several other obvious characteristic modes (123.8, 184.8, 218.9, 285, 357.2, 386.5, 407.7, 443.2 and 505.8 cm^−1^) were observed below 550 cm^−1^. These peaks are similar to fibrous red phosphorus characteristic modes [25,26], which indicated that parts of P_4_H_6_ were thoroughly dissociated when exposed to laser or decompression. At ambient pressure, phosphorus hydrides often undergo disproportionation into phosphorus-rich phosphanes upon exposure to light and heat [24]. However, we did not observe the Raman peaks from other phosphanes from the residue, which proves that P_2_H_4_ dimerized directly into P_4_H_6_ at high pressure, corresponding to this equation:

(2) \begin{equation*} {\rm{2}}{{\rm{P}}_{\rm{2}}}{{\rm{H}}_{\rm{4}}} \to {{\rm{P}}_{\rm{4}}}{{\rm{H}}_{\rm{6}}} + {{\rm{H}}_{\rm{2}}}. \end{equation*}

From the recovered sample, it is confirmed that P_2_H_4_ dimerizes at high pressure. However, as both Raman and IR signal disappeared at above 20 GPa, we could not get *in situ* high-pressure vibrational modes. Therefore, it might be possible that other compounds generated at high pressure, such as P_4_H_6_·H_2_, which may easily decompose back to P_4_H_6_ and H_2_ upon decompression.

Figure 2d shows that the Raman spectra of the residue quenched from 35 GPa. After decompression to 1 atm, typical black phosphorus modes were observed [29,30]. Therefore, P_4_H_6_ eventually decomposed into elemental phosphorus at 35 GPa. Hence, the corresponding reaction is as follows:

(3) \begin{equation*} {{\rm{P}}_{\rm{4}}}{{\rm{H}}_{\rm{6}}} \to 4{\rm{P + 3}}{{\rm{H}}_{\rm{2}}}. \end{equation*}

From the *in situ* high-pressure XRD (Fig. 2d), the typical diffraction rings of cubic phosphorus further confirmed the thorough decomposition of P_4_H_6_ at high pressure.

### Stoichiometric evolutions of PH_3_ at low temperature

The superconductivity of elemental phosphorus has already been studied both experimentally and theoretically [31–33]. The maximum *T*_c_ is about 9.5 K at 32 GPa before it decreases with pressure. Near 100 GPa, the *T*_c_ is about 4.3 K at 160 GPa, and no superconducting transition was detected in the temperature range from 4 to 40 K. The much lower *T*_c_ of phosphorus compared to 100 K indicates that PH_3_ or other phosphorus hydrides should be responsible for the superconductivity observed at 200 GPa in Drozdov's work. Since Drozdov *et al.* increased the pressure at low temperature (*T* <200 K) [15], we speculate that this discrepancy is due to the different experimental protocols used in these two works. Low temperature could hinder the polymerization/decomposition of phosphorus hydrides and secure phosphorus hydrides to much higher pressure.

**Figure 3.**
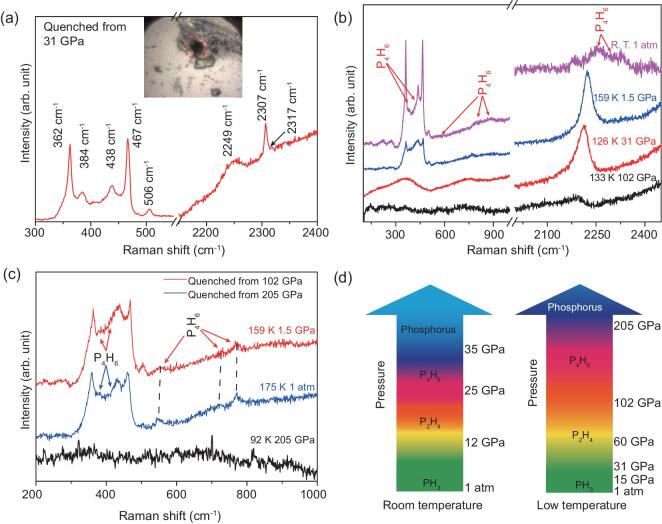
(a) The Raman spectrum of the sample quenched from 31 GPa. The inset image shows the photo-induced transition after laser irradiation. (b) The Raman spectra of the sample collected during decompression from 102 GPa. (c) The Raman spectra of the residue quenched from 205 (blue line) and 102 (red line) GPa, respectively. (d) The phase diagrams of PH_3_ at room temperature and low temperature.

To find out whether low temperature can hinder the reactions and further indentify the superconducting candidate, we studied the high-pressure behavior of PH_3_ at low temperature (<200 K). First, we compressed PH_3_ up to 31 GPa, when P_2_H_4_ dimerized into P_4_H_6_ at room temperature. However, after we decompressed the sample to ambient pressure at low temperature, the sample became transparent again (the inset image in Fig. 3a). As shown in Fig. 3a, the sample decomposed after laser irradiation and the resulting opaque solid (the inset image in Fig. 3a was identified as Hittorf's phosphorus) and two characteristic Raman modes from P_2_H_4_ (2307 and 2317 cm^−1^) were also found. The transparency of the residue and strong peaks from P_2_H_4_ suggest P_2_H_4_ was dominant in the sample decompressed from 31 GPa at low temperature. However, it can only survive below 25 GPa at room temperature, proving that low temperature can greatly hinder the polymerization of phosphorus hydrides. We further compressed the sample up to 60 GPa, and studied the Raman spectrum of the quenched residue. Similar photodecomposition and typical Hittorf's P Raman modes (Supplementary Fig. 5, available as Supplementary Data at *NSR* online) were observed, which further suggested that P_2_H_4_ could remain up to 60 GPa at low temperature.

As the superconductivity was observed at pressures >80 GPa, we compressed PH_3_ at low temperature up to 102 and 205 GPa, respectively, to investigate the responsible superconducting candidate. As shown in Fig. 3b and c, we did not observe any peaks from the Raman spectra at 102 and 205 GPa, due to its metallic state as identified by Drozdov *et al*. After decompressing to 31 GPa, a strong peak at around 2212 cm^−1^ was observed, and it shifted to around 2250 cm^−1^ after the sample was quenched to ambient conditions. We also observed several other peaks at around 383, 418, 798 and 880 cm^−1^, which agreed well with our simulated P_4_H_6_ (Supplementary Table 1, available as Supplementary Data at *NSR* online) Raman, confirming the residue recovered from 102 GPa and 133 K was P_4_H_6_. As shown in Fig. 3c, the Raman spectrum of the sample decompressed from 205 GPa is almost the same as that from 102 GPa, suggesting P_4_H_6_ could be stable up to 205 GPa at low temperature. As P_4_H_6_ was observed after decompression from 102 and 205 GPa, we propose that the corresponding superconducting candidate in Drozdov's work could be P_4_H_6_. By combining the PH_3_ structure evolutions at both room and low temperatures, we could obtain the phase diagram of PH_3_ under high pressure (Fig. 3d). As shown in Fig. 3d, at room temperature, two-step dimerization occurred at around 12 and 25 GPa, and P_4_H_6_ finally decomposed into elemental phosphorus at 35 GPa. However, at low temperature, P_2_H_4_ could exist up to 60 GPa. P_4_H_6_ was maintained from 102 to 205 GPa.

### Theoretical calculations

**Figure 4.**
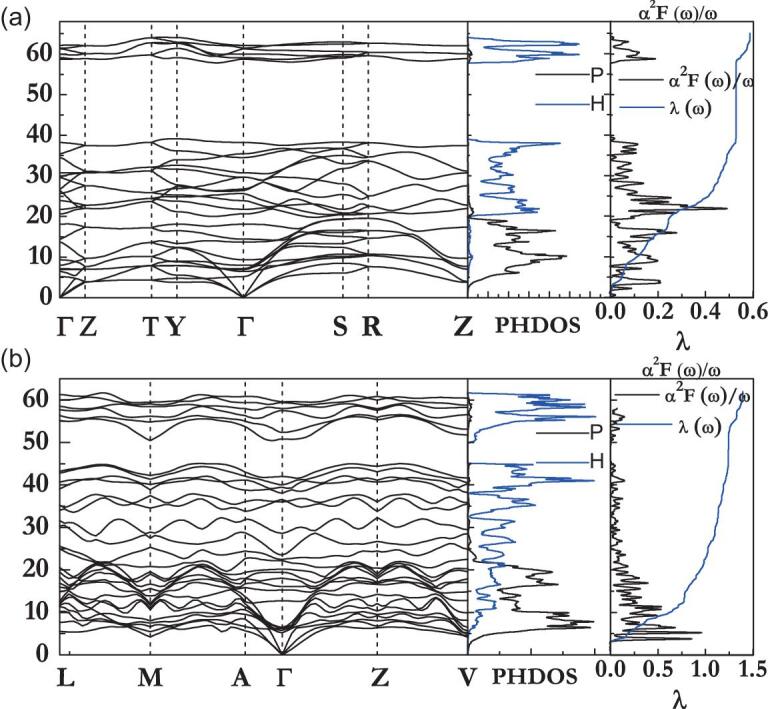
Phonon dispersions, phonon density of states projected onto atoms (PHDOS), the spectral functions *α*^2^F(*ω*)/*ω* and electron–phonon coupling integration of λ(*ω*) for the (a) Cmcm structure at 100 GPa and (b) C2/m structure at 200 GPa, respectively.

We further performed structural searches on P_4_H_6_ at 100, 150 and 200 GPa with maximum simulation cells up to 4 formula units (f.u.); two stable structures with space group Cmcm (<182 GPa) and *C*2/*m* (>182 GPa) were found. Phonon dispersions calculations of the two structures do not give any imaginary frequencies and therefore this verifies their dynamic stabilities (Fig. 4). The superconducting *T*_c_ was estimated using the Allen and Dynes modified McMillan equation [34] with a typical choice of ${\mathop \mu \limits}^* \, = \,0.13.$ The electron–phonon coupling constant λ of the Cmcm structure is only 0.59 (Table 1) at 100 GPa, and a superconducting *T*_c_ of 13 K was obtained. A relatively large λ value of 1.39 was found for the C2/m structure at 200 GPa, and the superconducting *T*_c_ was estimated to be 67 K. As summarized in Table 1, the estimated *T*_c_ agrees with the values measured by Drozdov *et al.*, suggesting that P_4_H_6_ could be responsible for the superconductivity.

**Table 1.** The calculated electron–phonon coupling constants (λ), the logarithmic average phonon frequency (*ω*_log_) and the *T*_c_ with μ* = 0.13.

Phases	Pressure (GPa)	λ	*ω*_log_	*T*_c_ (μ* = 0.13)
Cmcm	100	0.59	889	13 K
C2/m	200	1.39	700	67 K

Similar to H_2_S, PH_3_ is unstable at high pressure. Instead of becoming more hydrogen-enriching, it dehydrogenates through a series of polymerization/decomposition processes upon compression. This could be one of the critical factors that limit the maximum *T*_c_ near 100 K, at the same pressure where the H–S system has a *T*_c_ up to 180 K. These phenomena from H_2_S and PH_3_ highlight that avoiding the pressure-induced dehydrogenation or becoming more hydrogen-enriched is vital for a superconducting hydride with a high *T*_c_.

## CONCLUSION

In summary, we determined the stability of PH_3_ under high pressure. At room temperature, two steps of polymerization were obtained. P_2_H_4_ and P_4_H_6_ were the reaction products of the first and second step dimerization, respectively. Above 35 GPa, the generated P_4_H_6_ completely decomposed into elemental phosphorus. However, at low temperature, P_4_H_6_ could remain up to 205 GPa. Vibrational measurements and theoretical simulation confirmed the formation of P_2_H_4_ and P_4_H_6_, which enriches the phase diagram of the P–H system under high pressure. Our work proves that the P_4_H_6_ phase can be generated under high pressure and suggests that it might be responsible for the reported superconducting transition.

## METHODS

Solidified PH_3_ was prepared via a cryogenic method and sealed into a symmetric DAC at ∼2 GPa for our *in situ* high-pressure measurements. T301 stainless steel and tungsten gaskets were used for the room-temperature and low-temperature measurements, respectively. The ruby fluorescence and Raman shifts of the diamond were used to calibrate the pressure. A micro-Raman system (Renishaw, UK) with a 532-nm laser excitation was used to obtain the sample's Raman spectra. The high-pressure *in situ* IR spectra were collected on a Bruker VERTEX 70v FTIR spectrometer and a custom-built IR microscope. High-pressure XRD measurements were carried out at the BL15U1 beamline of the Shanghai Synchrotron Radiation Facility (λ = 0.6199 Å) [35]. Low temperature was generated by cryostat using liquid nitrogen. Detailed information about each cycle is provided in the Supplementary Materials, available as Supplementary Data at *NSR* online. The *ab initio* structure predictions for P_4_H_6_ were performed using the particle swarm optimization technique implemented in the CALYPSO code [36,37]. CALYPSO has been used to investigate many materials at high pressures [38–42]. The *ab initio* structure relaxations were performed using density functional theory with the Perdew–Burke–Ernzerhof generalized gradient approximation implemented in the Vienna *ab initio* simulation package (VASP) [43]. Details of the simulations are provided in the Supplementary Materials, available as Supplementary Data at *NSR* online.

## Supplementary Material

nwz010_Supplemental_File
