# Supplementary material for: Stoichiometric evolutions of PH3 under high pressure: implication for high-Tc superconducting hydrides
Source: Natl Sci Rev. 2019 Jan 22;6(3):524–31. doi: 10.1093/nsr/nwz010 (PMC8291478; doi:10.1093/nsr/nwz010)
Supplement: nwz010_Supplemental_File [file nwz010_supplemental_file.doc]

**Supporting Information**

**Pressure-induced dehydrogenation and superconductivity of phosphine**

Ye Yuan^#,1^, Yinwei Li^#,2^, Guoyong Fang,^#,3^ Guangtao Liu^1^, Cuiying Pei^1^, Xin Li^1,4^, Haiyan Zheng^1^, Yuexiao Pan^3^, Ke Yang^5^, and Lin Wang^*,1^

^1^Center for High Pressure Science and Technology Advanced Research (HPSTAR), Shanghai 201203, China

^2^School of Physics and Electronic Engineering, Jiangsu Normal University, Xuzhou 221116, China

^3^Key Laboratory of Carbon Materials of Zhejiang Province, College of Chemistry and Materials Engineering, Wenzhou University, Wenzhou 325035, China

^4^Department of Physics, Fudan University, Shanghai 200433, China

^5^Shanghai Institute of Applied Physics, Chinese Academy of Sciences, Shanghai 201203, People’s Republic of China

^#^Y. Y., Y. L. and G. F. contribute equally to this work

^*^To whom correspondence may be addressed. Email: wanglin@hpstar.ac.cn

**COMPUTATIONAL METHODS**

In the structural prediction, we have extensively searched the structure of P_4_H_6_ through CALYPSO (Crystal structure AnaLYsis by Particle Swarm Optimization).^1,2^ CALYPSO has been used to investigate a great variety of materials at high pressure.^3–6^ The fixed-composition structure search was considered converge when >1000 successive structures were generated after a lowest energy structure was found. *Ab initio* structure relaxations were performed using density functional theory (DFT) with the Perdew-Burke-Ernzerhof (PBE) generalized gradient approximation (GGA) implemented in the Vienna *ab* *initio* simulation package.^7^ Van der Waals density functional method^8–10^ was used in the optimization of selected structures. The all-electron projector augmented wave^11^ pseudopotentials with 3*s*^2^3*p*^3^ and1*s*^1^ valence configurations were choosed for P and H atoms, respectively. An energy cutoff of 700 eV and a Monkhorst-Pack Brillouin zone sampling grid with a resolution of 0.3 Å^-1^ were used in the structure searches. The low enthalpy structures found were then re-optimized with denser grids better than 0.2 Å^-1^ and a higher energy cutoff of 900 eV. The crystal structures were drawn using the VESTA software. Phonon dispersion and electron-phonon coupling (EPC) calculations were performed with density functional perturbation theory using the Quantum-ESPRESSO package^12^ with a kinetic energy cutoff of 90 Ry. 4×4×4 and 6×6×2 *q*-meshes in the first Brillouin zones were used in the EPC calculations for the Cmcm and C2/m structures, respectively. A same force convergence threshold (1 meV/Ǻ) for structural optimizations was applied to avoid any unnecessary errors caused by the use of two different codes.

**LOW TEMPERATURE MEASUREMENT**

Low temperature was generated by cryostat via using liquid nitrogen as cryogenic fluid bath. Detailed information about each cycle as follow:

Cycle 1: we firstly decreased the temperature to about 140 K and compressed the sample to 31 GPa at that temperature. After we quenched the sample to ambient pressure, we recovered the sample to room temperature and studied the sample via Raman spectroscopy.

Cycle 2: we firstly decreased the temperature to about 110 K and compressed the sample to 60 GPa at around that temperature. Then, we decompressed the sample to near 14 GPa. As the four screws have already totally loosed, we slowly increased the temperature to further decompress the sample. At 133 K, the pressure reduced to near 3 GPa. At room temperature, the pressure recovered to ambient pressure.

Cycle 3: we firstly decreased the temperature to about 140 K and compressed the sample to 102 GPa at around that temperature. Then, we decompressed the sample to near 30 GPa. As the four screws have already totally loosed, we slowly increased the temperature to further decompress the sample. At 159 K, the pressure reduced to near 2 GPa. At room temperature, the pressure recovered to ambient pressure.

Cycle 4: we firstly decreased the temperature to about 95 K and compressed the sample to 205 GPa at around that temperature. Then, we decompressed the sample to near 70 GPa. As the four screws have already totally loosed, we slowly increased the temperature to further decompress the sample. At around 140 K, the pressure reduced to near 35 GPa. At around 160 K, the pressure reduced to near 5 GPa. At around 170 K, the pressure reduced to near ambient pressure.


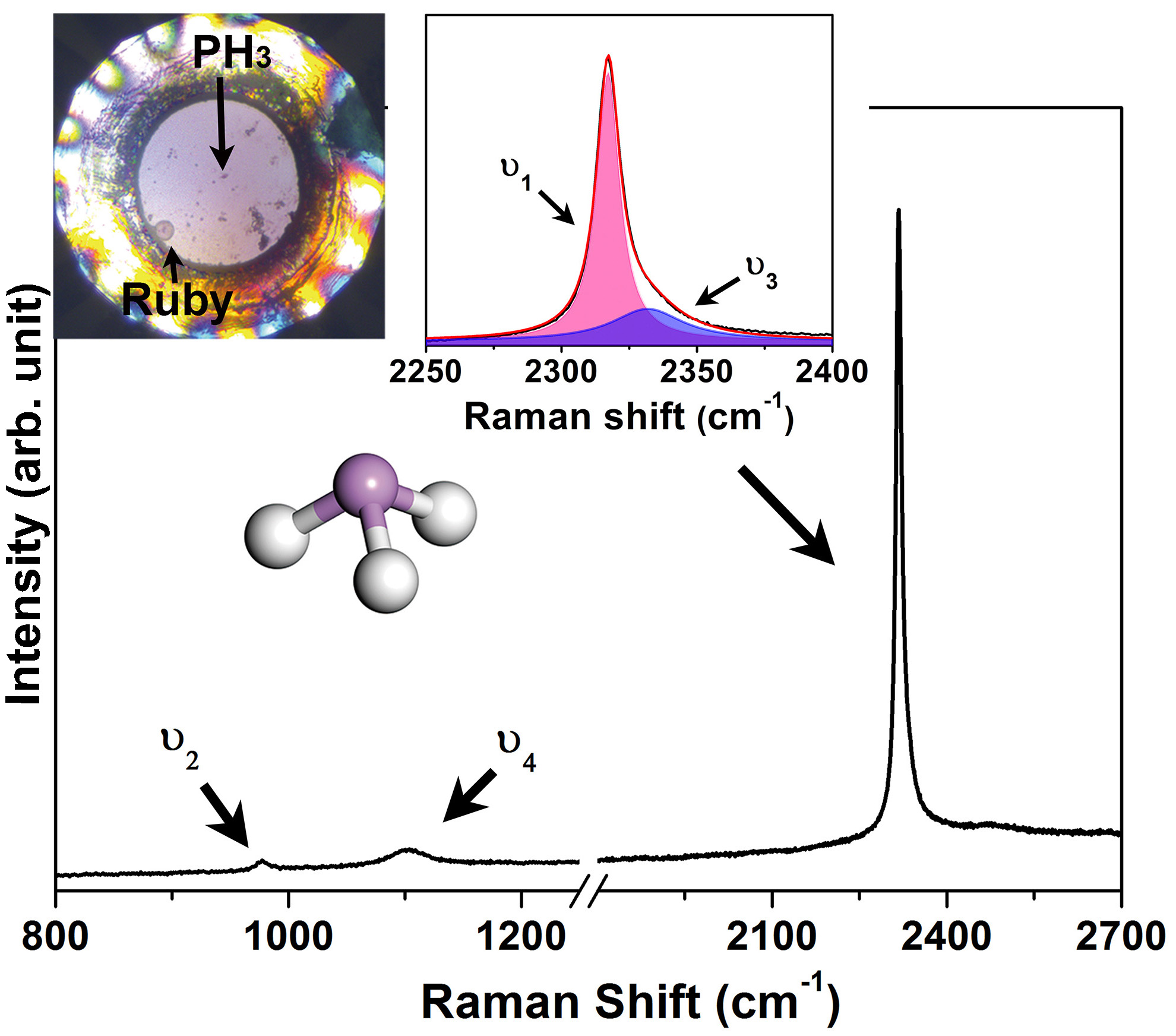


**Figure S1**. The Raman spectrum of the sample measured at 2.4 GPa. The inset is the optical micrograph of the corresponding sample and model of the PH_3_ molecule.


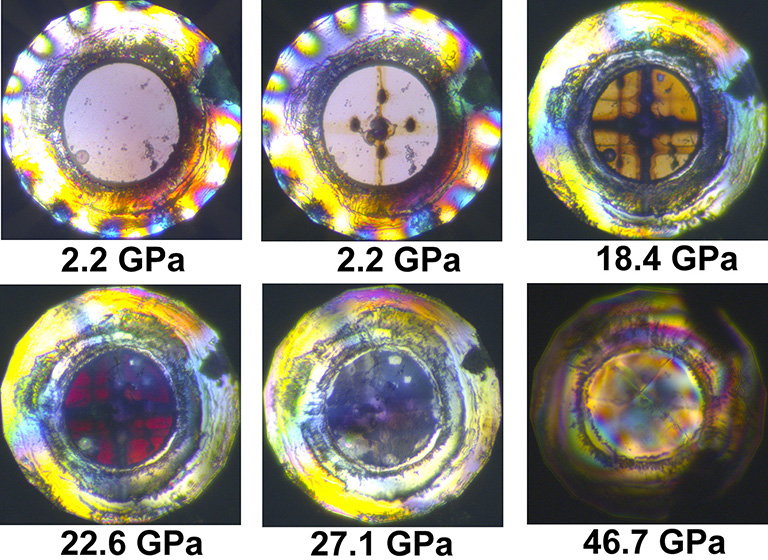
­­­­

**Figure S2.** Optical micrograph of the sample at 2.2, 18.4, 22.6, 27.1 and 46.7 GPa. The sample became opaque after x-ray irradiation.

**
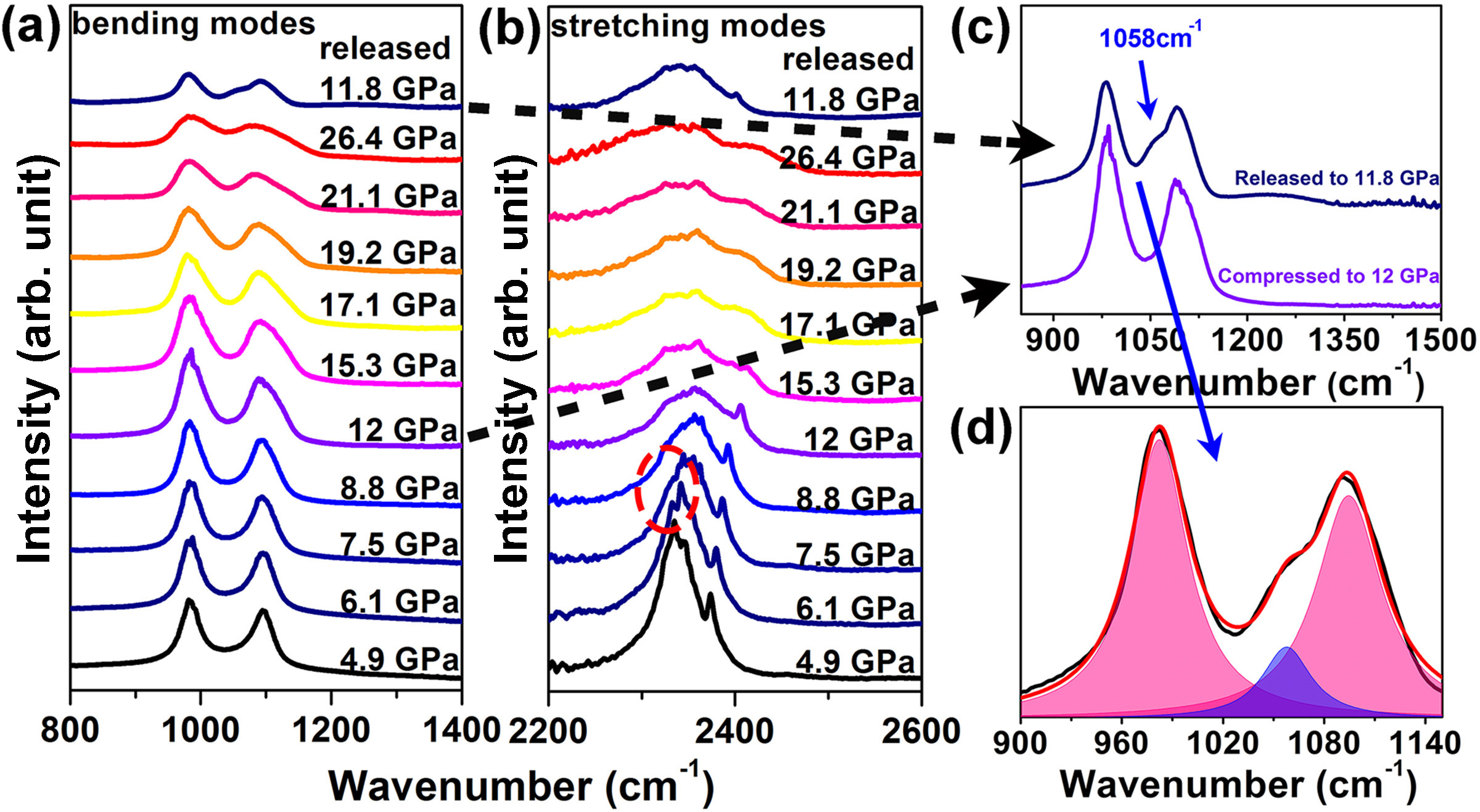
**

**Figure S3.** (a) and (b) The IR spectra of PH_3_ at various pressures. The new shoulder (2329 cm^-1^) was observed at 7.5 GPa (marked by red circle). (c) The IR spectra of PH_3_ measured at 12 (during compression) and 11.8 GPa (during decompression), respectively. (d) Multipeak Lorentz method was used to fit IR spectrum at 11.8 GPa during the decompression.

**
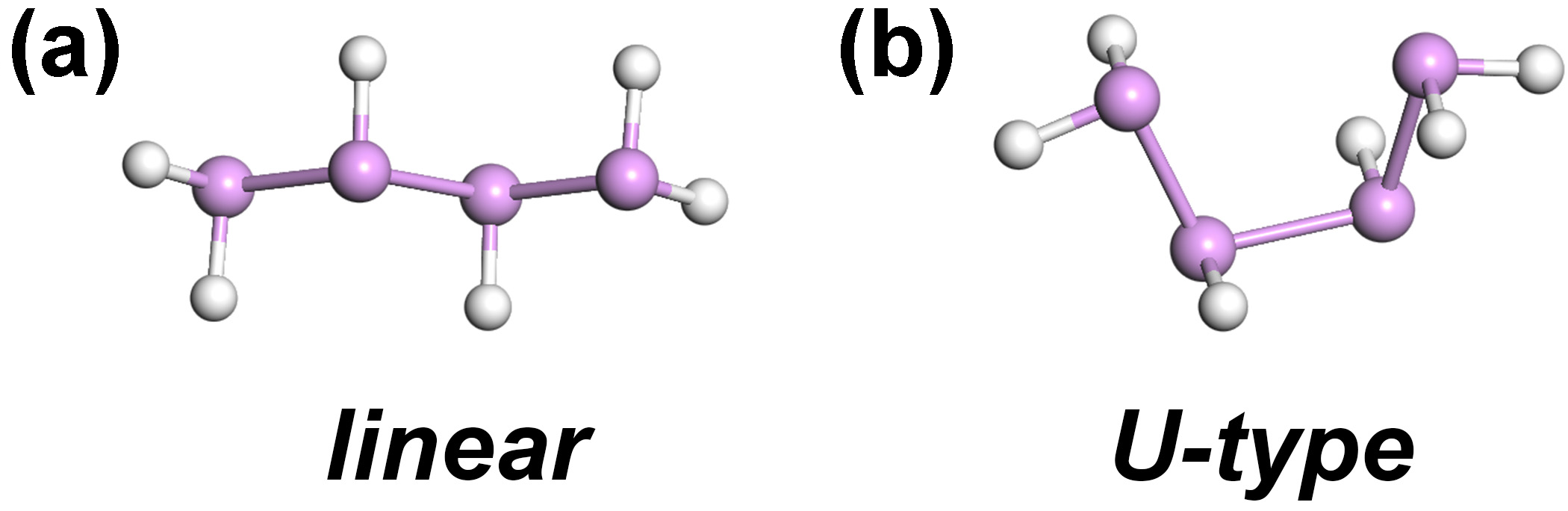
**

**Figure S4.** The structures of phosphorus hydride ((a) linear and (b) U-type P_4_H_6_).


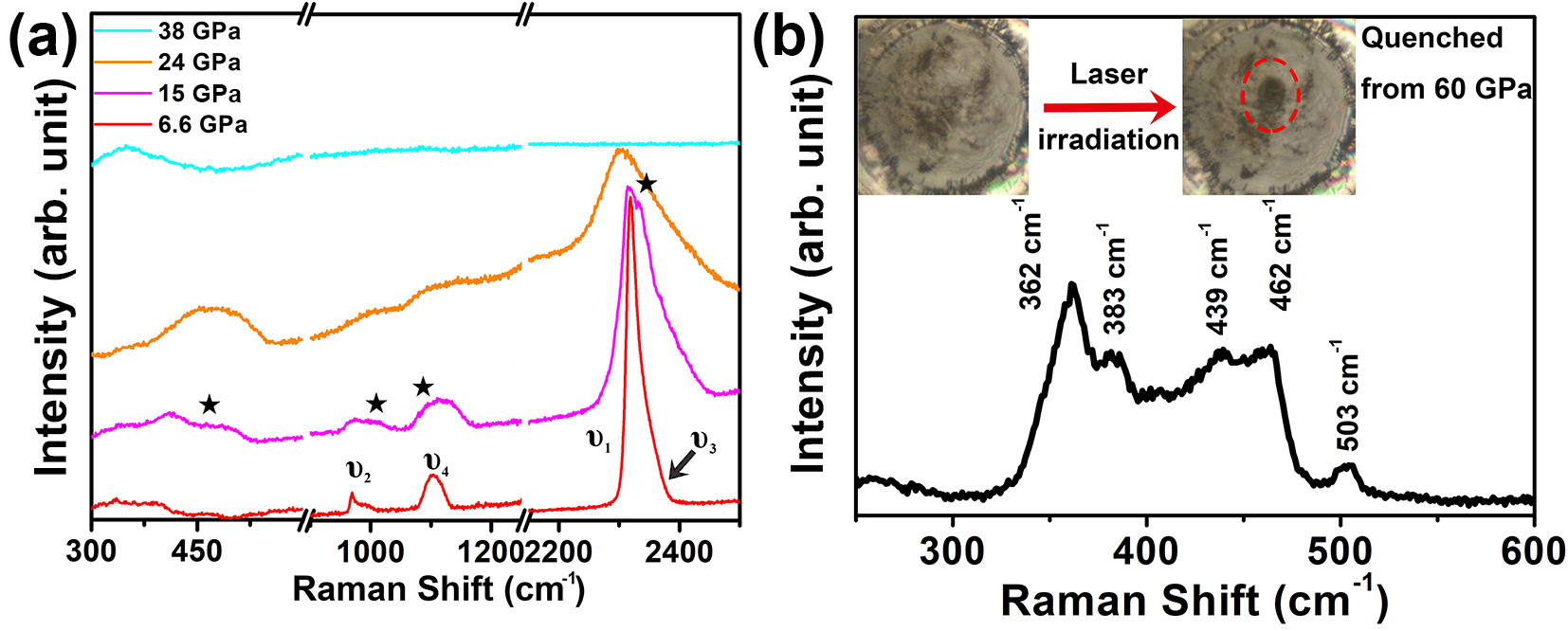


**Figure S5**. (a) *In situ* low temperature Raman spectra of PH_3_ at different pressure. At 15 GPa, several new peaks emerged, which indicated P_2_H_4_ generated. (b) The Raman spectrum of the Hittorf’s phosphorus transformed from the sample quenched from 30 GPa at low temperature. The inset optical images show the photo-induced transition of the residue before and after laser irradiation.


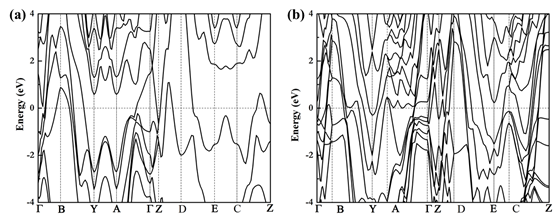


**Figure S6.** The calculated band structures of (a) the Cmcm structure at 100 GPa, and (b) the C2/m structure at 200 GPa. Both structures show metallic nature in view of several bands crossing the Fermi levels.

**Table S1** Simulated Raman of linear and U-type P_4_H_6_ in the gas phase.

| Number | Frequency | corrected*0.967 | Intensity(a.u.) | Assignment |
| --- | --- | --- | --- | --- |
| **Linear P_4_H_6_** |  |  |  |  |
| 1 | 20.0 | 19.3 | 0.0 |  |
| 2 | 75.2 | 72.8 | 0.0 |  |
| 3 | 84.0 | 81.3 | 1.7 |  |
| 4 | 107.8 | 104.3 | 0.0 |  |
| 5 | 158.1 | 152.9 | 9.7 |  |
| **6** | 402.8 | 389.5 | 41.2 | P-P stretching |
| 7 | 412.7 | 399.1 | 0.0 |  |
| 8 | 421.8 | 407.9 | 77.8 | P-P stretching |
| 9 | 625.1 | 604.5 | 0.0 |  |
| 10 | 629.8 | 609.0 | 0.5 |  |
| 11 | 637.3 | 616.3 | 0.0 |  |
| 12 | 721.8 | 698.0 | 0.0 |  |
| 13 | 772.0 | 746.6 | 12.9 |  |
| 14 | 867.4 | 838.8 | 21.3 |  |
| 15 | 894.8 | 865.2 | 0.0 |  |
| 16 | 910.5 | 880.4 | 19.5 |  |
| 17 | 1108.9 | 1072.3 | 75.7 | PH_2_ scissoring |
| 18 | 1109.7 | 1073.1 | 0.0 |  |
| 19 | 2355.7 | 2277.9 | 293.5 | PH symmetric stretching |
| 20 | 2365.1 | 2287.1 | 0.0 |  |
| 21 | 2373.9 | 2295.6 | 0.0 |  |
| 22 | 2374.0 | 2295.6 | 507.3 | PH_2_ symmetric stretching |
| 23 | 2385.3 | 2306.6 | 220.7 | PH_2_ asymmetric stretching |
| 24 | 2387.4 | 2308.6 | 0.0 |  |
| **U-type P_4_H_6_** |  |  |  |  |
| 1 | 56.0 | 54.2 | 1.3 |  |
| 2 | 67.0 | 64.7 | 0.8 |  |
| 3 | 87.1 | 84.2 | 4.5 |  |
| 4 | 130.2 | 125.9 | 0.9 |  |
| 5 | 176.3 | 170.5 | 9.5 |  |
| 6 | 364.4 | 352.4 | 52.3 | P-P stretching |
| 7 | 414.6 | 400.9 | 10.5 |  |
| 8 | 434.8 | 420.4 | 17.9 | P-P stretching |
| 9 | 589.1 | 569.7 | 2.1 |  |
| 10 | 639.6 | 618.5 | 0.9 |  |
| 11 | 650.5 | 629.0 | 0.7 |  |
| 12 | 730.6 | 706.5 | 8.1 |  |
| 13 | 742.6 | 718.1 | 15.1 |  |
| 14 | 835.4 | 807.8 | 7.9 |  |
| 15 | 895.5 | 865.9 | 21.4 |  |
| 16 | 945.9 | 914.6 | 7.7 |  |
| 17 | 1102.4 | 1066.0 | 61.8 | PH_2_ scissoring |
| 18 | 1104.8 | 1068.4 | 0.9 |  |
| 19 | 2362.4 | 2284.4 | 1.0 |  |
| 20 | 2365.7 | 2287.6 | 515.2 | PH_2_ symmetric stretching |
| 21 | 2372.0 | 2293.8 | 390.8 | PH symmetric stretching |
| 22 | 2381.2 | 2302.6 | 8.5 |  |
| 23 | 2387.3 | 2308.5 | 106.4 | PH_2_ asymmetric stretching |
| 24 | 2393.4 | 2314.4 | 0.1 |  |

**References**

[1] Wang, Y.; Lv, J.; Zhu, L.et al. *Comput. Phys. Commun.* **2012**, *183* (10), 2063, 2063–2070.

[2] Wang, Y.; Lv, J.; Zhu, L.et al. *Phys. Rev. B.* **2010**, *82* (9), 1-8.

[3] Li, Y.; Wang, L.; Liu, H. et al. *Phys. Rev. B* **2016**, *93* (2), 2-6.

[4] Li, Y.; Hao, J.; Liu, H. et al. *Sci. Rep.* **2015**, *5* (MAY), 9948.

[5] Li, Y.; Hao, J.; Liu, H. et al. *J. Chem. Phys.* **2014**, *140* (17).

[6] Lv, J.; Wang, Y.; Zhu, L. et al. *Phys. Rev. Lett.* **2011**, *106* (1), 19-22.

[7] Kresse, G.; Furthmüller, J. *Phys. Rev. B* **1996**, *54* (16), 11169-11186.

[8] Rydberg, H.; Dion, M.; Jacobson, N. et al. *Phys. Rev. Lett.* **2003**, *91* (12), 1-4.

[9] Román-Pérez, G.; Soler, J. M. *Phys. Rev. Lett.* **2009**, *103* (9), 1-4.

[10] Klime, J.; Bowler, D. R.; Michaelides, A. *Phys. Rev. B* **2011**, *83* (19), 1-13.

[11] Joubert, D. *Phys. Rev. B.* **1999**, *59* (3), 1758-1775.

[12] Scandolo, S.; Giannozzi, P.; Cavazzoni, C. et al. *Zeitschrift fur Krist.* **2005**, *220* (5–6), 574-579.
